# A hybrid machine learning approach for estimating the water-use efficiency and yield in agriculture

**DOI:** 10.1038/s41598-022-10844-2

**Published:** 2022-04-25

**Authors:** Hossein Dehghanisanij, Hojjat Emami, Somayeh Emami, Vahid Rezaverdinejad

**Affiliations:** 1 Agricultural Research, Education and Extension Organization, Agricultural Engineering Research Institute, Post Box 31585-845, Karaj, Alborz, Iran; 2 Department of Computer Engineering, University of Bonab, Bonab, Iran; 3 Department of Water Engineering, University of Tabriz, Tabriz, Iran; 4 Department of Water Engineering, Urmia University, Urmia, Iran

**Keywords:** Plant sciences, Environmental social sciences, Mathematics and computing

## Abstract

This paper introduces the narrow strip irrigation (NSI) method and aims to estimate water-use efficiency (WUE) and yield in apple orchards under NSI in the Miandoab region located southeast of Lake Urmia using a machine learning approach. To perform the estimation, a hybrid method based on an adaptive neuro-fuzzy inference system (ANFIS) and seasons optimization (SO) algorithm was proposed. According to the irrigation and climate factors, six different models have been proposed to combine the parameters in the SO-ANFIS. The proposed method is evaluated on a test data set that contains information about apple orchards in Miandoab city from 2019 to 2021. The NSI model was compared with two popular irrigation methods including two-sided furrow irrigation (TSFI) and basin irrigation (BI) on benchmark scenarios. The results justified that the *NSI* model increased WUE by 1.90 kg/m^3^ and 3.13 kg/m^3^, and yield by 8.57% and 14.30% compared to TSFI and BI methods, respectively. The experimental results show that the proposed SO-ANFIS has achieved the performance of 0.989 and 0.988 in terms of *R*^*2*^ criterion in estimating WUE and yield of NSI irrigation method, respectively. The results confirmed that the SO-ANFIS outperformed the counterpart methods in terms of performance measures.

## Introduction

Water resources are declining in many regions of the world. Due to climate change, increased air temperatures, and reduced precipitation, we will face a decline in water resources in the future^1, 2^. Iran is an arid and semi-arid region in terms of climate, the amount of rainfall, and the limitations of water resources in this region. Optimal use of available water resources is an important goal of water conveyance and distribution systems. Surface irrigation is one of the most common irrigation methods in the world. More than 95% of the agricultural land in Iran is currently under the surface irrigation method. Despite the complexity of this irrigation method, researchers and users have not paid much attention to it. The current efficiency of surface irrigation in Iran is estimated at less than 35%^1, 3, 4^. Surface irrigation is easy and needs inexpensive equipment to convey and distribute water in different areas. The maintenance and operation costs of the surface irrigation method are lower than other methods. Surface irrigation is performed according to topography and product type with different methods, including basin irrigation (BI) and two-sided furrow irrigation (TSFI). Due to the limited facilities for developing irrigated agriculture, increasing water-use efficiency by managing the irrigation and productivity of existing water and soil resources is necessary^4, 5^. Considering the excessive consumption of water resources, especially in the agricultural part of Lake Urmia, it is essential to precisely estimate the water-use efficiency (WUE) and yield by using optimal irrigation methods combined with artificial intelligence methods. WUE is an essential factor for identifying the adaptability of crops in water-limited regions under current climate conditions and future global changes^6–13^. In addition, yield prediction, particularly strategic products is an interesting research topic for agricultural meteorologists due to the importance of national and international economic planning.

In the recent decade, researchers have evaluated the yield and WUE in orchards according to irrigation management and different surface irrigation methods^14–18^. Osman^19^ stated that weak design and improper irrigation management in surface irrigation are the main reasons for low water-use efficiency. Lampinen *et al.*^20^ investigated soil and plant data and evapotranspiration for irrigation management of walnut trees in California, USA. Fernandes-Silva^21^ by examining the effect of different irrigation regimes (dryland irrigation with 30% and 100% water requirement) on yield and WUE of olive, reported that crop evapotranspiration (ETc) is the most influential parameter in changes in fruit yield. Dahikar and Rode^22^ proved that artificial neural networks (ANNs) have good efficiency in estimating crop yield. Dzikiti et al.^23^ estimated the water requirement of young and productive apple orchards in South Africa using the evapotranspiration model. Their findings confirmed that this model offers reasonable estimates in mature and young gardens. Emami and Choopan^24^ estimated barley yield using radial basis function (RBF) and feed-forward neural (GFF) models in Torbat Heydariyeh, located in southeastern Iran. The results showed that the RBF model with the input parameter of irrigation water levels could better estimate the barley yield. Bang et al.^25^ by comparing fuzzy logic, ARMA, SARIMA, and ARMAX methods, concluded that the fuzzy logic method has an excellent ability to predict crop yield. Kumar et al.^26^ used the random forest (RF) model to estimate crop yield. The results showed that the RF algorithm has a high capability in estimating crop yield by considering the minimum number of parameters. Sharifi^27^ proved that the Gaussian process regression algorithm has the best performance in estimating barley yield. Prasad et al.^28^ used the random forest algorithm for estimating cotton crops for the regional level. The results indicated that the RF model had a high potential in predicting crop yield. Dehghanisanij et al.^29^ reported irrigation-fertilizer and crop variety parameters are the most effective parameters in estimating yield and water productivity of tomato crops.

According to the above-mentioned studies, climatic factors are effective parameters affecting the yield and WUE. Given the complexity of the impact of climatic factors on plant growth, it is necessary to estimate its effects. In this paper, a new irrigation method referred to as narrow strip irrigation (NSI) was introduced to reduce applied water in apple orchards. Then, a hybrid predictive method based on adaptive neuro-fuzzy inference system (ANFIS) and season optimization (SO) algorithm is proposed to estimate WUE and yield. To summarize, the contributions o this paper are as follows:

- Introducing the narrow strip irrigation (NSI) method for the first time and estimate its WUE and yield parameters. The NSI method reduces the growth of weeds and prevents the penetration of water outside the shade of the tree.
- Introducing the hybrid SO-ANFIS method to estimate the WUE and yield parameters of the NSI irrigation method. The SO-ANFIS takes the advantages of both SO algorithm and ANFIS methods.
- Evaluating the SO-ANFIS method on a benchmark dataset and compared it with state-of-the-art WUE and yield estimation methods. The results justify that the proposed SO-ANFIS outperformed its counterparts in terms of performance measures.

The remaining part of this paper is organized as follows. Section “Materials and methods” describes the test case and the working principle of the proposed approach. In section “Results and discussion”, the results and discussions are presented. Section “Conclusion” concludes the paper and presents some suggestions for future work.

## Materials and methods

### Test case

**Figure 1:**
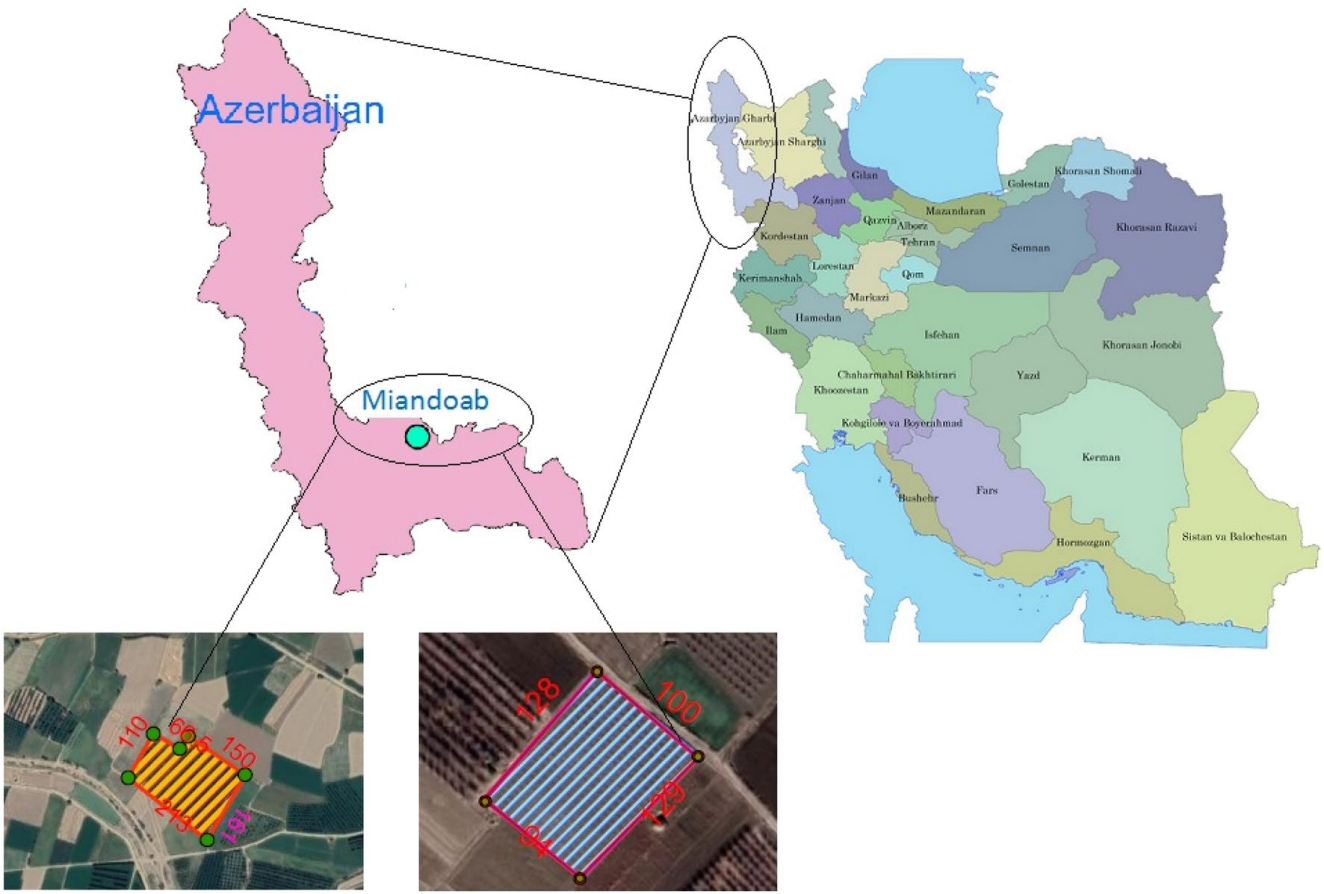
Study area on the map, the graph is plotted in in ESRI ArcGIS Desktop 10.8.2.28388 software [https://www.esri.com/en-us/arcgis/about-arcgis/overview].

This study was conducted in the agricultural lands of Dolatabad village located in Miandoab region. Miandoab is a city in the northwest of Iran located in the southeast of Lake Urmia. The geographical coordinates of Miandoab are 46° 2′ N and 36° 58′ E at 1314 m above sea level (Fig. 1). In this region, the weather is variable, with relatively hot summers and cold winters. Miandoab is a significant agricultural region in West Azerbaijan province. The main crops in Miandoab are wheat, barley, sugar beet, corn, and apple orchards.

### Field studies and sampling

In this study, a total of 120 field data from two farms under study (M_1_ and M_2_ farms in Fig. 1) were collected. This data set was randomly divided into two parts; 80% of the data was used for model training and the remaining 20% for tests. Soil sampling was performed from the end of the tree shading surface and three depths of 0–30 cm, 30–60 cm, and 60–90 cm. Three types of irrigation methods including BI, NSI, and TSFI, were considered. The cultivars studied were *Golden Delicious*. The distances of the trees were 6 × 6 m^2^. The dimensions of the control and treatment strips were 3.6 × 6 m^2^ and 6 × 6 m^2^, respectively. The irrigation interval was considered equal to 10–15 days based on the climate condition to ensure an optimal outcome. The crop was harvested on September 30, 2021.

#### Water-use efficiency

WUE can be defined as (Eq. 1)^30^.

1 $$WUE = \frac{{Y\,({\text{usually}}\,{\text{economical}}\,{\text{yield}})}}{{{\text{applied}}\,{\text{water }}(P_{e} + \, I + \, SW)}}$$

where $Y$ denotes the economical yield was measured base on the delivered product to the market, $I$ is irrigation water measured using a WSC flume, *P*_*e*_ is effective rainfall and $SW$ indicates soil water depletion from the root zone during the growing season. The *SW* is estimated based on the water balance at the selected farm.

Improving the economic water use efficiency at the farm level requires better adaptation and coordination of water use according to the needs of products at the time and amount of its use, which ultimately improves crop yield. This is possible by using new emerging technology and applying better management methods. Applying new management methods in planning for planting, irrigation, and using other inputs plays an effective role in achieving high WUE. Chemical and physical analyses of soil and fertilizers used are presented in Tables 1, 2 and 3.

**Table 1:** Physical and chemical analysis of soil at a depth of 0–90 cm.

B.D (gr/cm^3^)	PWP (cm^3^/cm^3^)	FC (cm^3^/cm^3^)	OC (%)	TNV (%)	θ_s_	pH	Texture	EC (ds/m)	Silt	Sand	Clay	Depth (cm)
1.25	0.14	0.328	1.35	10.4	52	8.3	Sic	0.875	52	9	39	0–30
1.25	0.149	0.331	0.82	14.9	57	8.11	Sic	1.45	46	11	43	30–60
1.25	0.144	0.328	-	17.8	55	8.05	Sic	2.78	48	11	41	60–90

EC: Electrical Conductivity; pH: Acidity of water; θ_s_: Saturated moisture (volumetric); TNV: Lime; OC: Organic carbon; FC: Field capacity; PWP: Permanent wilting point; B.D: Soil Bulk Density.

**Table 2:** Physical and chemical characteristics of the soil.

K_R_ (kg/ha)	P_R_ (kg/ha)	N_R_ (kg/ha)	K_A_ (ppm)	P_A_ (ppm)	N_T_ (%)	Date of harvest	Date of planting	Variety	Cultivation pattern	Area (ha)
100	75	50	158	4.8	8	2021/09/30	23 year	Red/golden	Apple	0.8

N_T_: Total nitrogen; P_A_: Absorbable phosphorus; K_A_: Absorbable potassium; N_R_: Nitrogen requirement; P_R_: Phosphorus requirement; K_R_: Potassium requirement.

**Table 3:** Amounts of fertilizer used in an apple orchard.

Fertilizer	Amount	Date
Di-ammonium phosphate	60 kg	2020/03/26
Ammonium sulfate	50 kg	2020/03/26
Potassium sulfate	100 kg	2020/03/26
Zinc sulfate	30 kg	2020/03/26
Iron sulfate	50 kg	2020/03/26
Agricultural sulfur	100 kg	2020/03/26
Thiobacillus bacteria	5 kg	2020/03/26
Manure	5 ton	2020/03/26

#### Application efficiency

Application efficiency (AE) indicates losses in the farm in the form of deep infiltration and runoff at the end of the farm. At each irrigation interval, AE is calculated as follows^43^:

2 $$AE = \frac{{D_{z} }}{{ \, D_{app} }} \times 100$$

where *D*_*z*_ shows the average water storage in the root zone depth (mm) and *D*_*app*_ is the average depth of water entering the irrigated area. *D*_*app*_ is defined as

3 $$D_{app} = \frac{V}{ \, A}$$

where *V is* the volume of inlet flow to the irrigated area (lit) and *A* indicates the irrigated area (m^2^).

### Irrigation methods

#### Narrow strip irrigation

**Figure 2:**
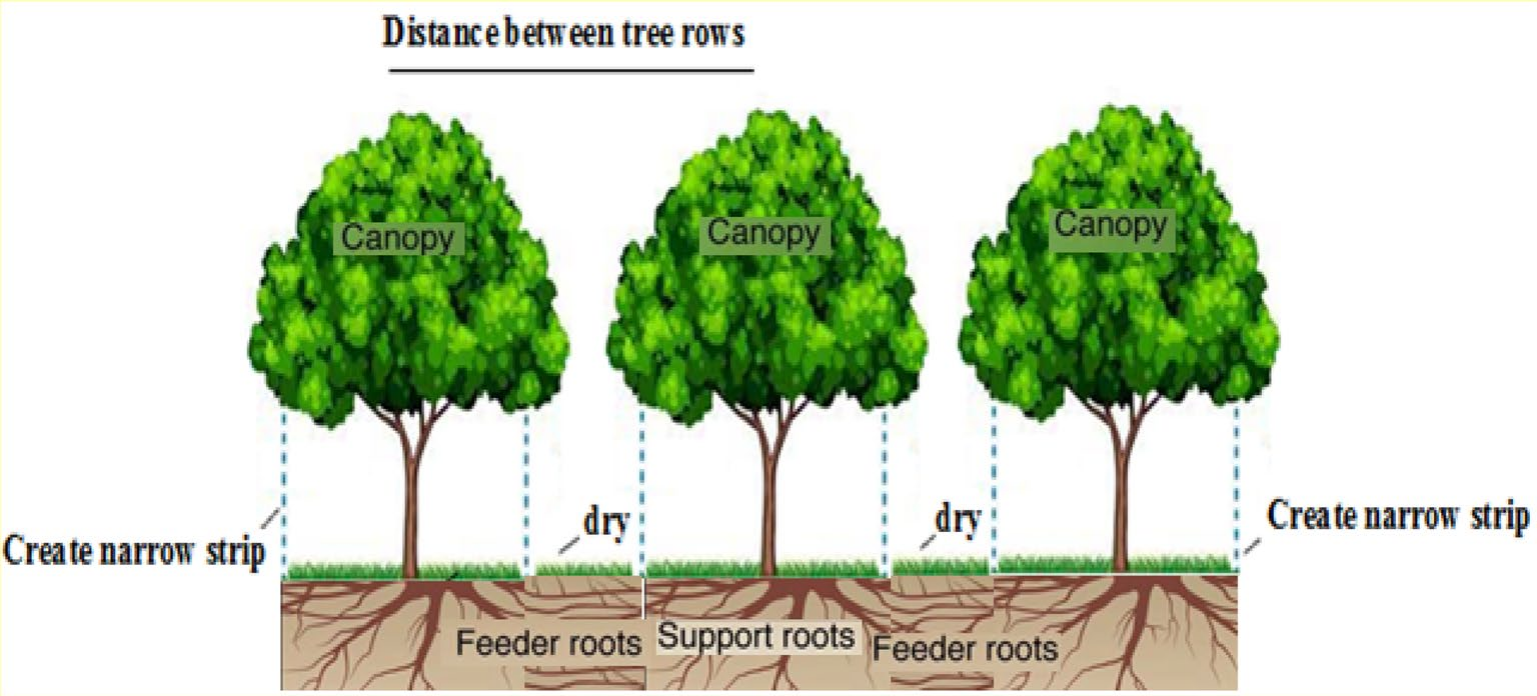
Schematic of NSI method.

In the NSI method, the entire orchard surface is not irrigated, and evaporation losses are minimized. Therefore, the daily water requirement of the tree is mainly limited to the amount of transpiration from the aerial parts exposed to sunlight. Plant shading level is one of the critical factors in calculating the water requirement of trees. This parameter is determined experimentally in terms of the type and age of the plant (between 50 and 70%)^31^. Overall, in the *NSI*, the main area where transpiration occurs is the shading level. In the NSI method (Fig. 2), a space is created in the middle of the trees row in orchards. This area is dry during irrigation, and applied water is reduced due to the lack of weed growth and water evaporation from this area. In the NSI, the following equation is used to calculate the daily water requirement of the plant^31^:

4 $$R_{r} = {\mkern 1mu} ET_{c} = \left( {h_{s} + 0.15(1 - \, h_{s} )} \right){\mkern 1mu}$$

where $R_{r}$ is the maximum daily water requirement (mm/day), $ET_{c}$ is the maximum daily evapotranspiration (mm/day), and $h_{s}$ is the maximum shading level (%). According to the studies, the shading level for trees is 50–70% in the optimal state^31^. In this study, the shading level of apple trees was determined based on age and crown environment.

#### Two-sided furrow irrigation

**Figure 3:**
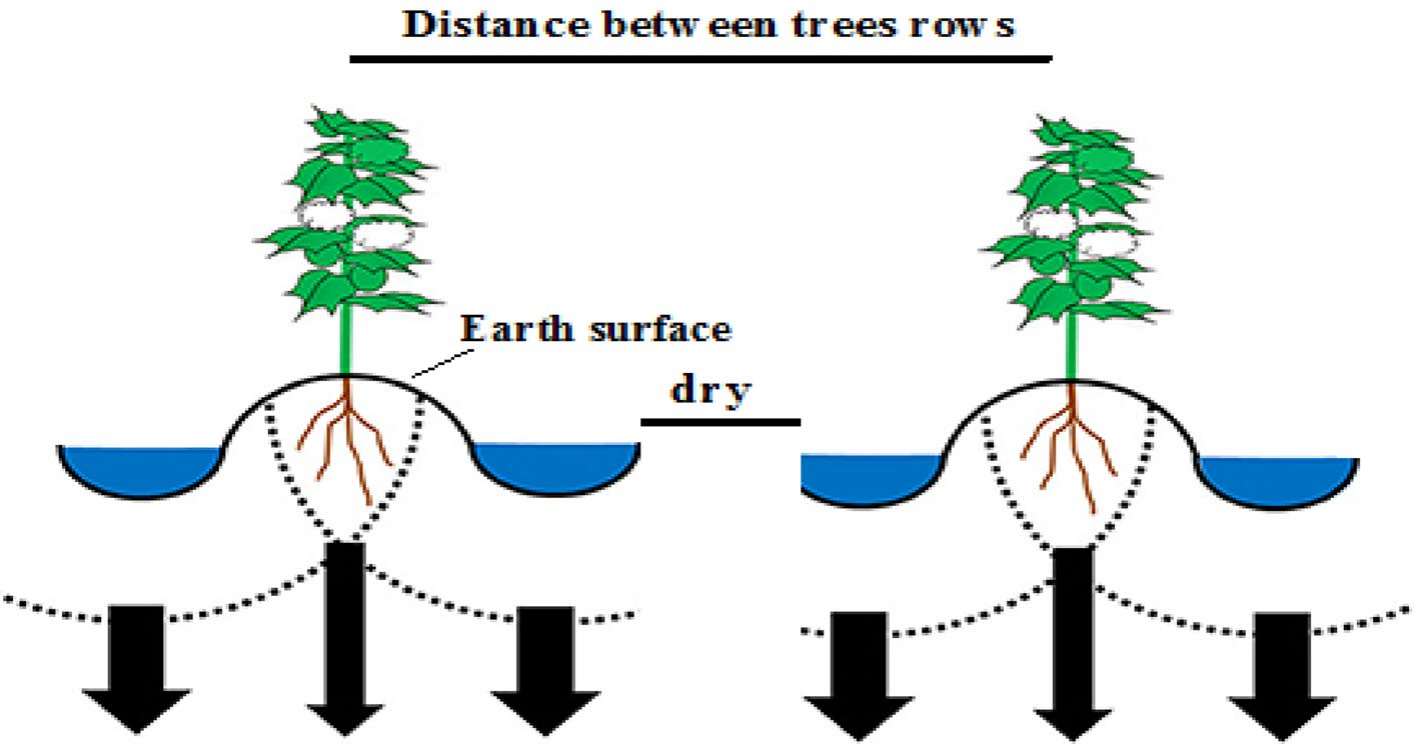
Schematic of TSFI method.

In the TSFI method, water moves inside the furrow on both sides of the trees and deep in the soil irrigates the root development area vertically and laterally (Fig. 3). This method tries to wet the soil surface less. The water is directed by two furrows created on either side of the rows of trees. The distance of the furrows from the rows of trees varies depending on the distance between the rows of trees, soil texture, and age of the trees. By performing furrow irrigation, two dry parts are created in the orchard. These arid areas form one along the rows of trees and the other between the furrows in the middle of the rows of trees. Try to prevent weeds from growing in dry areas as much as possible with tools such as garden tractors, cultivators, or retractors. In fact, the existence of these arid areas and the lack of weed growth and water evaporation from these arid areas, which play a role in the real reduction of water consumption. If these two arid areas are full of weeds, water consumption will not be really saved and only the irrigation efficiency will increase due to the movement of water in the furrows^32^.

#### Basin irrigation

**Figure 4:**
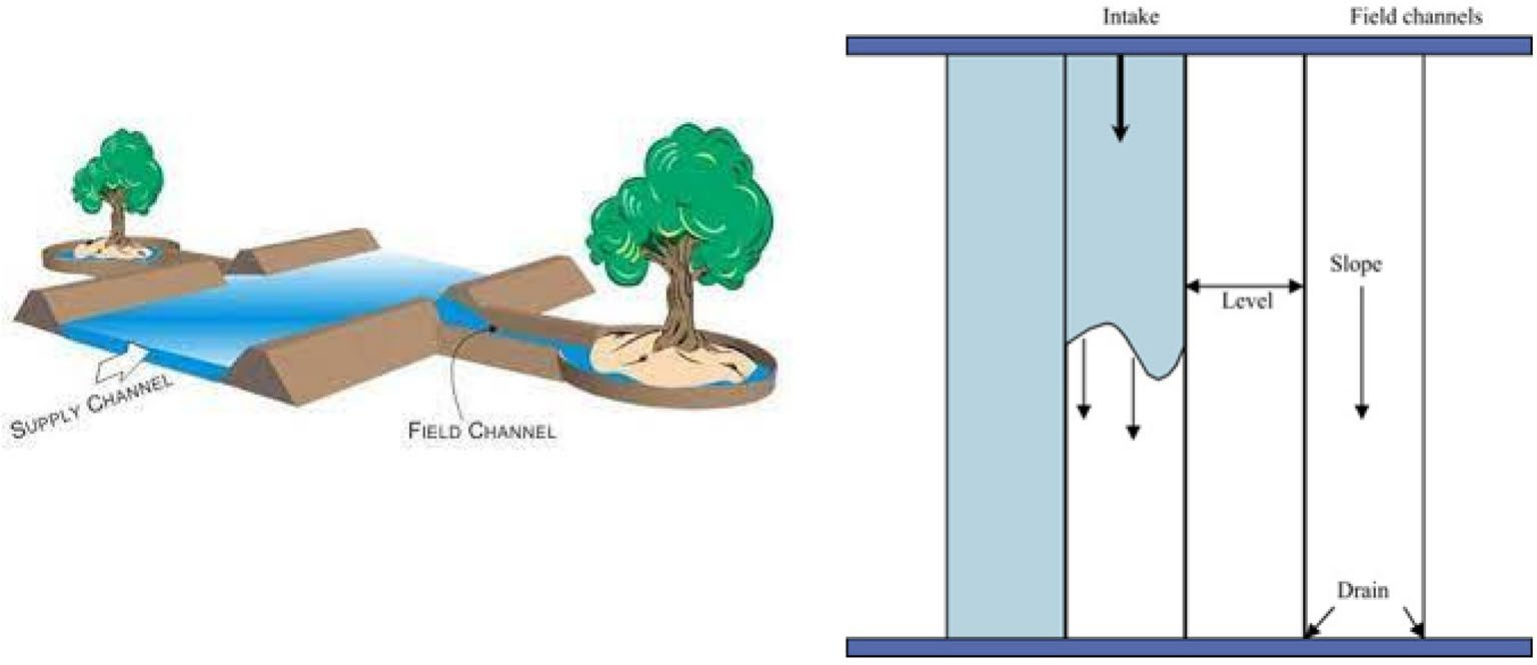
Schematic of BI method.

BI is a method in which water penetrates the soil permanently or intermittently, and the soil is permanently submerged (Fig. 4). In basin irrigation, water penetrates the crown area of the plant, and the problem of clogging heavy soils and reducing soil aeration occurs^32^.

In general, in the NSI method, compared to the TSFI and BI methods, the water in the shade of the tree travels in a straight path at the same width and travels to the next tree. In this method, water penetration is prevented outside the shade of the tree, and conditions for weed growth will not be provided.

### Season's optimization (SO) algorithm

**Figure 5:**
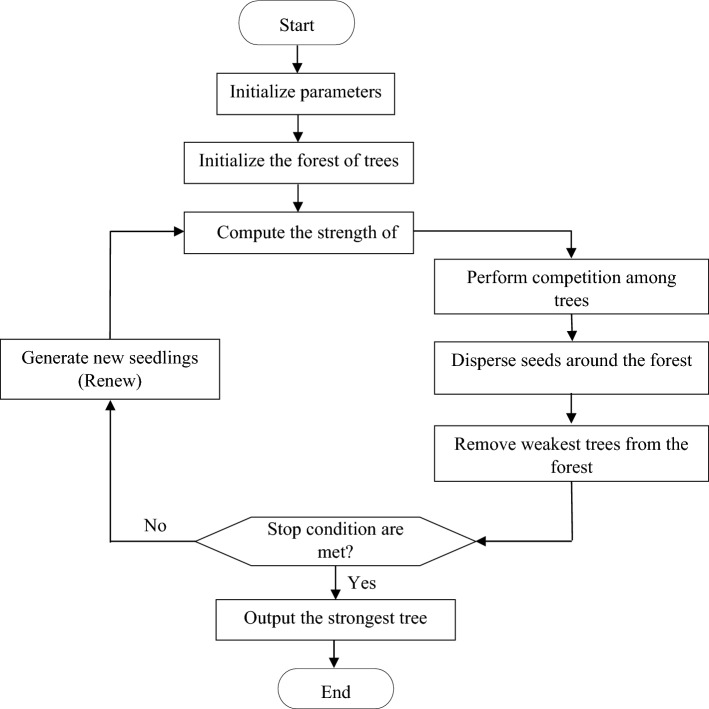
Flowchart of the SO algorithm.

The SO algorithm is a population-based optimization meta-heuristic^33^. It models the growing process of trees in four seasons of a year. Figure 5 illustrates the flowchart of the SO algorithm. The SO is an iterative algorithm in which each agent is called a tree. For solving an optimization problem, the algorithm starts its process with a population referred to as a forest. Each member of the population is called a tree which denotes a potential solution for the given problem. For an optimization problem $f(X) = f(x_{1} ,x_{2} ,...,x_{D} )$ with *D* dimensions, the initial forest *F* is initialized as follows^33^:

5 $$\begin{aligned} F & = [T_{1} ,T_{2} ,...,T_{N} ] \\ T_{i} & = [t_{i1} ,t_{i2} ,...,t_{iD} ] \\ t_{ij} & = l_{ij} + r_{ij} \cdot (u_{ij} - l_{ij} ) \\ \end{aligned}$$

where is $r_{ij}$ a random number in the interval [0, 1] generated by the uniform distribution $u_{ij}$ and $l_{ij}$ are the upper and lower bounds of $t_{ij}$, respectively. The fitness of each tree is evaluated by a strength function.

6 $$S_{i} = S(T_{i} ) = S(t_{i1} ,t_{i2} ,...,t_{iD} )$$

The algorithm updates the trees using four operators, including renew, competition, seeding, and resistance. The renew phase models the impact of the spring on the growth of trees. The following equations are defined to model the renew phase mathematically:

7 $$\begin{aligned} F^{y + 1} & = \{ F^{y} \} + \{ R\} \\ R & = \Phi (p_{r} \times A^{y} ) \\ \end{aligned}$$

where *R* indicates the set of new seedlings, $F^{y}$ shows the forest at the *y*th iteration, $A^{y}$ is the number of seeds generated in the previous autumn and $p_{r}$ is the renew rate. The function $\Phi$ randomly produces some seedlings in various locations of the forest. The algorithm does not execute the renew phase in the generation *y* = 0.

The competition phase modes the growth of trees in the summer. In this phase, the trees compete with their neighbor trees on shared resources, including nutrients, water, light, and other resources. To simulate the competition process, first $N_{c}$ most vital trees are identified. The number of neighbors of a cored tree is calculated as follows^33^:

8 $$\begin{aligned} N_{c} & = \left\lceil {p_{c} \times N} \right\rceil \\ Z_{i} & = \left\lceil {\tau_{i} \times N_{g} } \right\rceil \\ N_{g} & = (N - N_{c} ) \\ \end{aligned}$$

$\tau_{i}$ is the normalized fitness of *T*_*i*_, which is calculated as follows:

9 $$\tau_{i} = (S_{i} - \min (I))/\left( {\sum\limits_{k = 1}^{N} {S_{k} } } \right),\;\;\;I = \left\{ {S_{k} |k = 1,\;2,\; \ldots ,\;N} \right\}$$

Then, $Z_{i}$ neighbors are elected to create the neighborhood zone. To simulate the impact of the competition on a neighbor $T_{i}$, the below relationship is defined:

10 $$\begin{aligned} & T_{j}^{y + 1} = \frac{1}{{\Lambda_{j} + 1}} \times \varphi (T_{j}^{y} ) \\ & {\text{where}} \\ & \varphi (T_{j}^{y} ) = T_{j}^{y} + \theta \\ & \Lambda_{j} = \sum\limits_{k = 1}^{{Z_{i} }} {S_{k} \times \Delta_{j,k}^{ - 2} \times \lambda_{j,k} } \\ & \Delta_{j,k} = \sqrt {\sum\limits_{z = 1}^{D} {(T_{jz} - T_{kz} )^{2} } } \\ & \lambda_{j,k} = \left\{ {\begin{array}{*{20}l} 1 \hfill & {{\text{if}}(S_{k} \ge S_{j} )} \hfill \\ {1 - \gamma } \hfill & {else} \hfill \\ \end{array} } \right. \\ \end{aligned}$$

where $T_{j}^{y}$ is the location of $T_{j}$ in the generation *y*. $\Lambda_{j}$ is the value of competition index or crowdedness, which computes the effect of the neighbors on $T_{j}$. *D* shows the number of variables of trees. The function $\varphi (.)$ calculates the growth of $T_{j}$ in the same environment when its neighbors are ignored. $S_{k}$ indicates the strength/ fitness of the *k*th neighbor tree, $\Delta_{j,k}$ is the distance between $T_{j}$ and the *k*th neighbor, the variable $\lambda_{j,k}$ is the effect of the neighbor on the growth of the tree $T_{j}$. The parameter $\gamma \in [0,1]$ is a random asymmetry index, which shows the value to which the impact of relatively weak neighbor is decreased^33^.

The new location of the cored tree $T_{i}$ is calculated as

11 $$T_{i}^{y + 1} = \left\{ {\begin{array}{*{20}c} {T^{*} } & {{\text{if}}\;\;S(T_{i}^{y} ) \le S(T^{*} )} \\ {T_{i}^{y} } & {{\text{if}}\;\;S(T_{i}^{y} ) > S(T^{*} )} \\ \end{array} } \right.$$

where $T^{*}$ shows the strongest neighbor tree around $T_{i}$.

The seeding phase is inspired by the seeding mechanism of trees in the autumn. In this phase, several trees are randomly selected and participate in the seeding phase. The number of seeds (*A*) at each generation is calculated as

12 $$A = \psi (p_{s} \times N)$$

where $p_{s}$ indicates the seeding rate, which is a uniform random number. The $\psi$ function identifies the fittest trees from the population. From each tree $T_{i}$ selected in the seeding phase, several elements are randomly identified, and their current values are updated with new random deals in the boundary of search space. Let *m* be a random number, and $\{ t_{i1} ,t_{i2} ,...,t_{im} \}$ are the elements selected from $T_{i}$, where $m < D$. Each component $t_{ij} \in T_{i}$ is calculated as

13 $$t^{\prime}_{j} = t_{j} + \ell \times r$$

$\ell$ is a two-valued variable, either 1 or − 1, and $r \in [l_{j} ,\;u_{j} ]$ is a random number.

The resistance phase simulates the resistance of the trees against harsh winter cold. The resistance operator removes the least-strength trees from the population. This operator is mathematically modeled as follows:

14 $$\begin{aligned} F^{y + 1} & = \{ F^{y} \} - \{ W\} \\ W & = \chi (p_{w} \times N) \\ p_{w} & = 1 - (1 - p_{s} ) \\ \end{aligned}$$

where *W* is the collection of weak trees. $\chi (.)$, removes $p_{w} \times N$ trees from the forest, $p_{s}$ is the resistance rate.

When the stopping measures are met, the algorithm updates the trees in the population by iteratively applying to renew, competition, seeding, and resistance operators. Finally, the fittest tree is identified as the optimal solution^33^.

### Adaptive neuro-fuzzy inference system (ANFIS)

The ANFIS integrates the artificial neural networks (ANNs) and fuzzy inference system (FIS)^34^. The ANFIS combines the advantages of both FIS and ANNs. The ANFIS system has high adaptation and fast learning capacity, captures the non-linear structure of processes, and causes less memorization. These characteristics make the ANFIS the best choice for predictive problems such as WUE and yield estimation problems. The ANFIS has been used successfully in various fields including mechanical design problems, chemical processes, data mining applications, communications, economics, geotechnical engineering problems, scheduling problems, and many other engineering problems.

**Figure 6:**
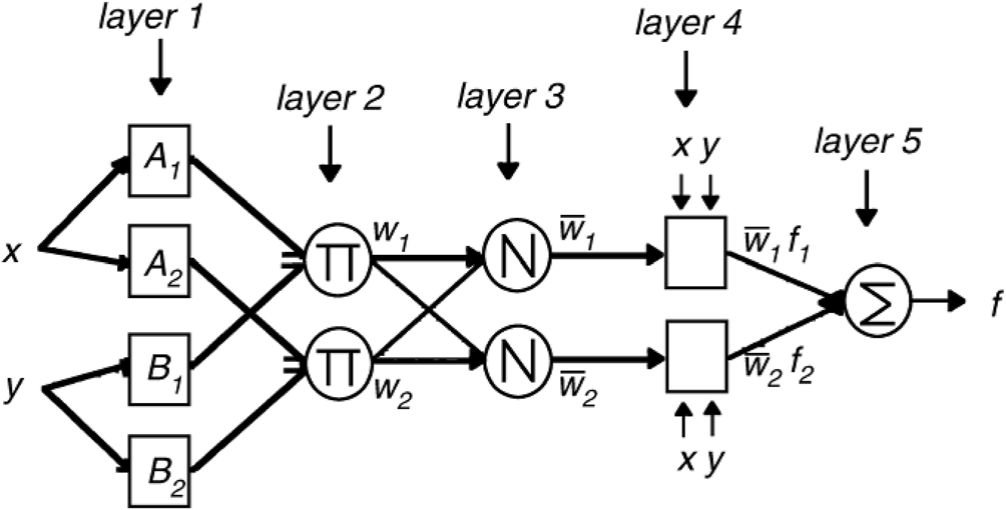
A big picture of the ANFIS system with two inputs.

In ANFIS, the relationship between inputs and outputs and the best values for the parameters related to the membership functions are identified by the fuzzy section and ANNs, respectively. The structure of ANFIS is determined considering the input data, rules, functions of the output membership function, and the membership degree. The ANFIS system with five layers is shown in Fig. 6. In the first layer, the level of dependence of each input data on different fuzzy domains is determined. The weight of the rules is obtained by multiplying the input values of each node in the second layer. The computation of the importance of regulations is carried out in the third layer. The rules layer is created by performing operations on the input signals described by the fourth layer. The network output is indicated by the fifth layer. ANFIS has *n* rules and *m* input components. Each rule $R_{i}$ is represented as follows:

15 $$R_{i} {\text{: if (}}x_{1} {\text{ is }}q_{i1} ){\text{ and (}}x_{2} {\text{ is }}q_{i2} )\,\,\,{\text{and}}\,\,...\,\,\,(x_{m} \,\,{\text{is}}\,\,\,q_{im} )\,\,\,{\text{then}}\,\,{\text{output = }}\,f_{i}$$

where $x_{j} \,$ indicates the *j*th input $q_{ij}$ indicates the membership function of the rule on $x_{j} \,$, *f*_*i*_ is the output of rule, The output of the network is presented as follows:

16 $$f(x) = \frac{{\sum\limits_{i = 1}^{n} {\mu_{i} f_{i} } }}{{\sum\limits_{i = 1}^{n} {\mu_{i} } }}$$

where $\mu_{{\text{i}}}$ indicates the activation degree of the rule. Each node has a function with adjustable parameters. $\mu_{{\text{i}}}$ is defined as follows:

17 $$\mu_{{\text{i}}} { = }\prod\limits_{j = 1}^{m} {q_{ij} } (x_{j} )$$

In the current implementation of ANFIS, we used the Gaussian membership functions, which is defined as follows:

18 $$q_{ij} (x) = \exp \left[ {\frac{1}{2}\left[ {\frac{{x - c_{ij} }}{{\sigma_{ij} }}} \right]^{2} } \right]$$

where $c_{ij}$ and $\sigma_{ij}$ are the center and standard deviation of the Gaussian membership function, respectively. Gaussian membership function is a popular method for specifying fuzzy sets because of its smoothness and concise notation. Five factors should be determined in designing ANFIS, the number and type of input and output fuzzy sets, the number of iterations, and the optimization method. The SO algorithm was used to optimize the parameters of the ANFIS membership function. In this paper, the fuzzy c-means clustering (FCM) is used to create fuzzy inference system which obtained superior results in the literature.

### SO-ANFIS model

Two structural parameters of the ANFIS system are antecedent and consequent parameters. For tuning these parameters, researchers often used gradient-based techniques. The main drawback of the gradient-based methods is the low convergence rate and trapping in local optima. Meta-heuristic algorithms can be used as efficient alternatives to overcome the limitations of gradient-based methods in training the ANFIS model. To train the ANFIS system using the SO algorithm, two issues need to be determined: strength function and the boundary of variables. In this study, root means square error (*RMSE*) is used as a strength function for evaluating the performance of the ANFIS system. Assume the following relationship:

19 $$\begin{aligned} {\text{R}}_{{\text{i}}} & {\text{: if }}I_{r} {\text{ is }}q_{1} {(}\sigma_{1i} ,\,c_{1i} ){\text{ and }}T_{emp} {\text{ is }}q_{2} {(}\sigma_{2j} ,\,c_{2j} )\,\,{\text{and}}\,\,P_{e} \,\,{\text{is }}q_{3} {(}\sigma_{3l} ,\,c_{3l} )\,{\text{ and}} \\ & \quad RH_{avg} {\text{ is }}q_{4} {(}\sigma_{4k} ,\,c_{4k} )\,\,\,{\text{and }}S_{sh} {\text{ is }}q_{5} {(}\sigma_{5t} ,\,c_{5t} ){\text{ then}} \\ y_{i} & = s_{1} I_{r} + s_{2} T_{emp} + s_{3} P_{e} + s_{4} RH_{avg} + s_{5} S_{sh} + s_{6} \\ \end{aligned}$$

**Figure 7:**
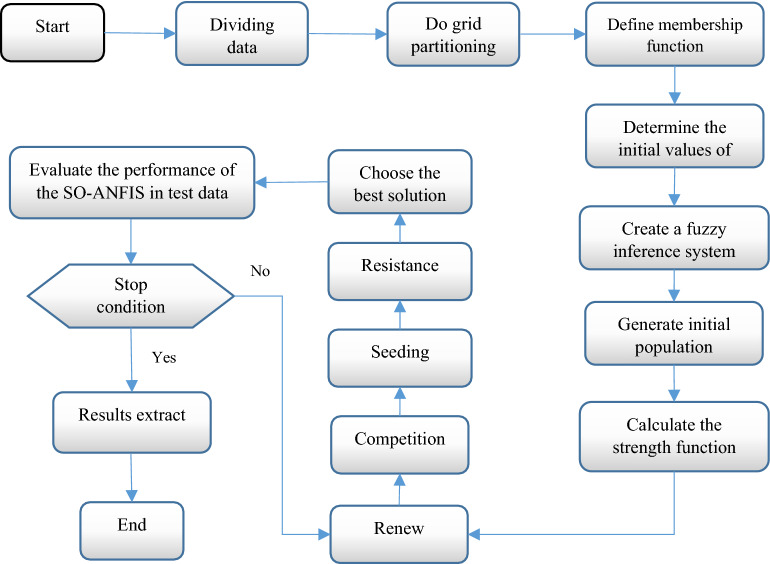
Flowchart of the SO-ANFIS.

The input variables are water consumption during the growing season (*Ir*), temperature (*T*_*emp*_), average relative humidity (*RH*_*avg*_), the amount of solar radiation in terms of sunshine hours (*S*_*sh*_), and the rainfall (*P*_*e*_) of each month of the growing season. The model parameters that need to be configured are $\sigma ,\,c,\,s_{1} ,\,s_{2} ,\,s_{3} ,s_{4} ,s_{5} ,s_{6}$. The variables $s_{1} ,\,s_{2} ,\,s_{3} ,s_{4} ,s_{5} ,s_{6}$ are consequent parameters, which should be measured during the ANFIS training process. The optimal values for parameters $c$ and $\sigma$ is measured by the SO algorithm. To identify the value of parameters $c$ and $\sigma$, first, a forest composed of several trees is initiated. Each tree contains candidate values for the ANFIS parameters. The trees are updated iteratively using four operators (renew, competition, seeding, and resistance). This process iterates for a pre-determined number of generations (Fig. 7).

### Performance Criteria

In the present study, *R*^*2*^, RMSE, SI, *δ*, and NSE indices were applied to appraise the ability of the introduced hybrid method^35^:

20 $$R^{2} = \left[ {\frac{{\mathop \sum \nolimits_{i = 1}^{n} \left( {w_{i} - \overline{w} } \right)(z_{i} - \overline{z} )}}{{\mathop \sum \nolimits_{i = 1}^{n} \sqrt {(w_{i} - \overline{w} )^{2} } \mathop \sum \nolimits_{i = 1}^{n} \sqrt {(z_{i} - \overline{z} )^{2} } }}} \right]^{2}$$

21 $$RMSE = \sqrt {\frac{1}{n}\sum\limits_{i = 1}^{n} {\mathop {(\mathop w\nolimits_{i} - \mathop z\nolimits_{i} )}\nolimits^{2} } }$$

22 $$SI = \frac{RMSE}{{\overline{w} }}$$

23 $$\delta \% = \frac{{\sum\limits_{i = 1}^{n} {\left| {(\mathop z\nolimits_{i} - \mathop w\nolimits_{i} )} \right|} }}{{\sum\limits_{i = 1}^{n} {\mathop w\nolimits_{i} } }} \times 100$$

24 $$NSE = 1 - \frac{{\sum\limits_{i = 1}^{n} {(w_{i} - \mathop z\nolimits_{i} )^{2} } }}{{\sum\limits_{i = 1}^{n} {(w_{i} - \overline{z} )^{2} } }}$$

$w_{i}$ and $z_{i}$ are the observed and predicted values of yield and WUE, respectively. $\overline{w}$ and $\overline{z}$ are average observed and predicted values of yield and WUE, respectively.

## Results and discussion

### Field monitoring

**Figure 8:**
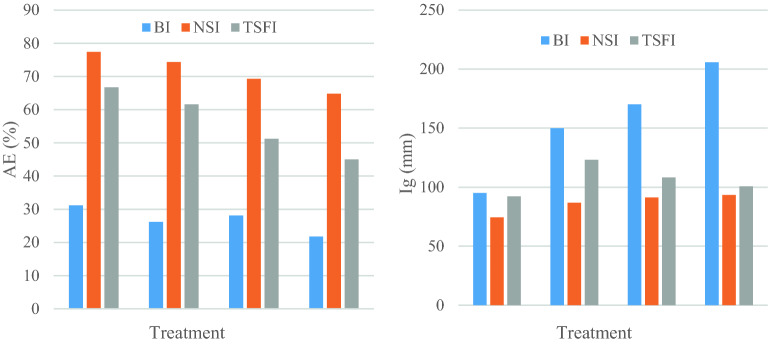
Comparison of application efficiency (AE) and Irrigation depth (Ig) in different treatments BI, NSI, and TSFI.

Table 4 and Fig. 8 present the results of irrigation depth, inlet flow, net irrigation requirement, and AE in research treatments. The first irrigation has the lowest WUE due to the dryer soil surface and impacts of tillage operations. Deep penetration losses are primarily due to the excellent permeability of the soil. The application efficiency increased to 21.4% due to the NSI method and irrigation time management compared to the two-sided irrigation method. Irrigation depth increased in treatment BI from the first to fourth irrigation event because of the loss of large amounts of water as deep infiltration and the scarcity of soil moisture increases at the point of root access. By increasing the irrigation depth, application efficiency decreased accordingly.

**Table 4:** Mean valuesof irrigation characteristics in research treatments.

Irrigation	Average inlet flow (L/s)			Cut-off time (min)			Irrigation depth (I_g_) (mm)			Net irrigation requirement (I_n_) (mm)			Application efficiency (AE) %		
	BI	NSI	TSFI	BI	NSI	TSFI	BI	NSI	TSFI	BI	NSI	TSFI	BI	NSI	TSFI
1	8.71	8.61	7.25	150	93	110	95	74.3	129	29.9	57.5	78.9	31.2	77.4	66.7
2	10.30	8.62	9.26	200	82	94	149.8	86.7	123.1	27.8	64.4	79.5	26.2	74.3	61.6
3	10.63	8.65	9.23	220	80	90	170.2	91.2	108.4	33.3	65	73	28.1	69.3	51.2
4	10.53	8.15	9.15	250	68	80	205.7	93.3	100.6	44.8	71.6	55.5	21.8	64.8	45

**Figure 9:**
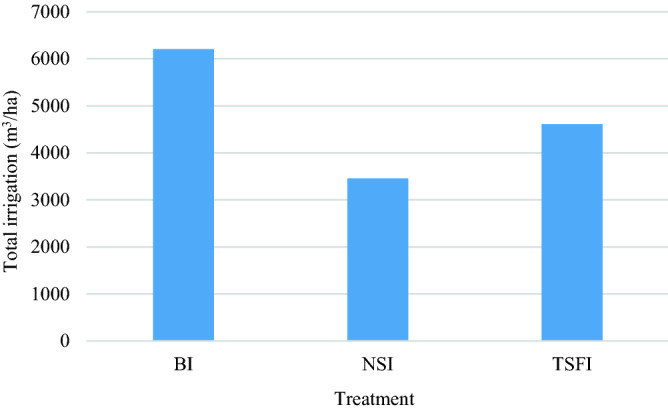
Comparison of total applied water in different treatments.

Based on the results, the average increase in application efficiency in NSI compared to BI is about 62.40%. The amount of applied water in the NSI method was 3455 m^3^/ha, which indicates a reduction of 42.80% and 22.70% of applied water in NSI treatment compared to BI and TSFI treatments (Fig. 9). The decreases were mainly attributed to the less soil surface wetted area, which was minimum in NSI and NSI < TSFI < BI.

The estimated yield in BI, NSI, and TSFI treatments was 30,000 kg/ha, 35,000 kg/ha, and 32,000 kg/ha, respectively. Higher yield in NSI and TSFI attributed to the soil moisture condition. The yield of NSI and TSFI treatments compared to the BI treatment was 14.30% and 6.25%, respectively, and in comparison with each other, increased by 8.57% (treatment NSI compared to treatment TSFI) (Table 5). The estimated WUE in NSI and TSFI treatments was 7.14 kg/m^3^ and 5.24 kg/m^3^, respectively (Table 5).

**Table 5:** Yield and WUE in research treatments.

Reduction of applied water (%)			WUE (kg/m^3^)			Yield (kg/ha)		
BI	NSI	TSFI	BI	NSI	TSFI	BI	NSI	TSFI
-	42.8	22.7	4.01	7.14	5.24	30,000	35,000	32,000

### Effect of soil properties

The results of descriptive statistics are shown in Table 6. Based on^36^ classification, the variation coefficient (CV), less than 15% shows low changes, between 15 and 35% moderate changes, and more than 35% great changes. According to this classification, soil sand content, tree age, and irrigation interval have moderate changes, soil acidity has low changes, and other variables have great changes (due to management factors). Absorbable phosphorus concentration at a depth of 30–60 cm in the soil is the most influential parameter for crop yield. The results of the present study are consistent with^37, 38^ studies. Elimination of tree irrigation at different growth periods reduces the quality and quantity of crop yield. Sedaghati et al.^28, 37^ concluded that increasing the irrigation interval from 25 to 45 days increased the percentage of porosity. According to studies, water flow and irrigation interval have a positive effect on crop yield^38^. Therefore, reducing the irrigation interval with methods such narrow strip^38^ can be considered as one of the management methods. Increasing the percentage of sand reduces the soil's ability to retain water and nutrients used by the plant. The age of the trees in this area is high, and with the increase of the tree's age, its ability to grow and produce its production gradually decreases.

**Table 6:** The results of descriptive statistics.

Variable	Unit	Min	Max	Avg	SD*	CV
Yield	kg/ha	25,000	35,000	30,000	5.89	1767.20
EC_0–30_	dS m^−1^	0.850	2.80	1.32	2.75	0.03
pH_0–30_	–	8.00	8.50	8.10	0.53	0.042
K_0–30_	ppm	120.00	158.00	132.00	3.75	4.80
P_0–30_	ppm	4.00	4.80	4.30	2.82	0.11
Clay_0–30_	–	30.00	39.00	33.00	3.22	1.10
Silt_0–30_	–	46.00	52.00	48.00	1.72	0.78
Sand_0–30_	–	7.50	9.00	8.10	3.15	0.25
EC_30–60_	dS m^−1^	1.35	1.45	1.38	0.82	0.011
pH_30–60_	–	7.80	8.11	8.00	0.92	0.075
K_30–60_	ppm	78.30	168.00	137.20	15.83	21.30
P_30–60_	ppm	3.30	4.10	3.70	1.72	0.058
Clay_30–60_	–	39.00	43.00	40.40	1.36	0.52
Silt_30–60_	–	40.00	46.00	42.00	1.86	0.74
Sand_30–60_	–	9.00	11.00	10.10	4.81	0.45
EC_60–90_	dS m^−1^	2.10	2.78	2.35	4.42	0.098
pH_60–90_	–	7.80	8.05	7.90	0.51	0.038
K_60–90_	ppm	123.00	179.30	131.40	2.40	3.02
P_60–90_	ppm	2.80	3.92	3.36	6.05	0.202
Clay_60–90_	–	38.00	41.00	39.00	0.96	0.37
Silt_60–90_	–	42.00	48.00	44.00	2.15	0.77
Sand_60–90_	–	9.00	11.00	10.10	4.81	0.45
EC_water_	dS m^−1^	0.98	1.12	1.00	0.82	0.008
Q**	L/s	8.09	8.78	8.17	0.40	0.03
Ii'	day	10.00	25.00	15.00	0.13	1.81
Area	ha	0.80	0.80	0.80	–	–
Ir″	m^3^/ha	3950.00	4945.20	4202.20	2.72	89.35
El.^^^	m	1300.00	1314.00	1306.00	0.19	2.42
Tree age	year	15.00	25.00	20.00	9.00	1.80

*Standard deviation, **Water flow, ′Irrigation interval, ″Amount of water consumed, ^^^Elevation.

### Modeling results

#### Investigating the effect of input combinations

Yield and WUE of apple trees depend on various factors, including water consumption during the growing season (*Ir*), climatic factors including temperature (*T*_*emp*_), average relative humidity (*RH*_*avg*_), the amount of solar radiation in terms of sunshine hours (*S*_*sh)*_, and the rainfall (*P*_*e*_) of each month of the growing season [http://tatweather.areeo.ac.ir/?LRef=52c6c899-7597-412d-83d7-c4cd2d05204b] (Table 7)^39, 40^.

**Table 7:** Effective input combination in estimating WUE and yield.

Model	Inputs parameters
ω1	Ir, P_e_, RH_avg_, T_emp,_ S_sh_
ω2	Ir, P_e_, RH_avg,_ S_sh_
ω3	Ir, P_e_, T_emp,_ S_sh_
ω4	Ir, P_e_, RH_avg_, T_emp_
ω5	Ir, T_emp_, RH_avg_, S_sh_
ω6	T_emp_, P_e_, S_sh_

**Figure 10:**
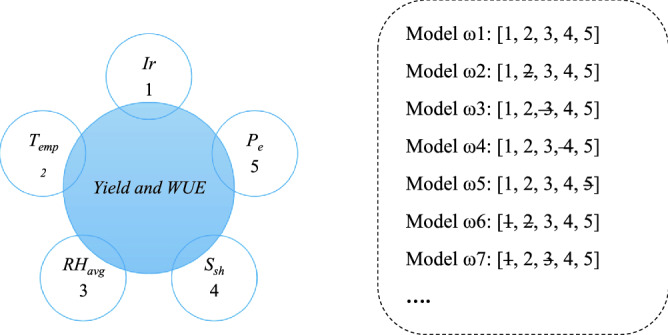
Some combinations of input parameters to estimate yield and WUE parameters.

To examine the most appropriate input parameters, different input combinations of parameters were evaluated. To select the most effective input parameters, first, all input combinations are considered to train the ANFIS model, and then the effective input combinations are selected. Next, ignore the remaining parameters one by one from the input combinations and train the model with the same structure and the rest ignored. This approach is also used by other researchers as given in references^39, 40^. Figure 10 shows 7 of the best-performing models.

The results obtained by the proposed SO-ANFIS method using different input combinations are shown in Table 8. Psize and fitness function evaluations (FEs) in the SO-ANFIS are considered 50 and 3000, respectively. According to the results in the observed data, the model *ω*2 obtained the most accurate results. The irrigation parameter (*Ir*) was proposed as the influential input variable in estimating yield and WUE. Then, rainfall and sunshine hours are essential, respectively. Sensitivity analysis showed that after irrigation and rainfall parameters, which affect leaves and plant reproductive growth, sunshine hours also play an important role in estimating yield. Montazer et al.^38^, Zeinadini et al.^40^, and Emami and Choopan^24^. Also stated that the amount of water consumed has an influential effect on crop yield .

**Table 8:** The efficiency of the proposed model in yield estimation.

Model	Train					Test					Membership functions	
	R^2^	RMSE	SI	δ	NSE	R^2^	RMSE	SI	δ	NSE	c	σ
ω1	0.980	0.008	0.010	0.878	0.962	0.960	0.009	0.012	1.160	0.915	0.4	0.2
**ω2**	**0.992**	**0.004**	**0.006**	**0.836**	**0.987**	**0.988**	**0.006**	**0.007**	**0.860**	**0.982**	**0.5**	**0.1**
ω3	0.975	0.010	0.014	1.125	0.918	0.972	0.012	0.018	1.415	0.906	0.7	0.2
ω4	0.890	0.012	0.016	1.142	0.846	0.851	0.020	0.033	1.650	0.705	0.5	0.1
ω5	0.905	0.013	0.018	1.325	0.823	0.890	0.017	0.025	1.480	0.740	0.3	0.2
ω6	0.880	0.015	0.026	1.589	0.725	0.868	0.019	0.031	1.620	0.710	0.1	0.1

Bold numbers indicate better results compared with others.

**Figure 11:**
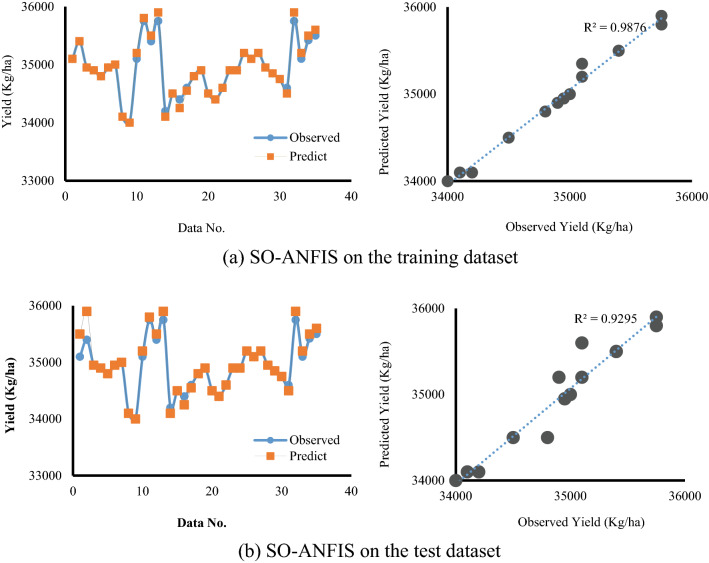
Comparison of predicted yield with observed results in the training and test stages.

**Figure 12:**
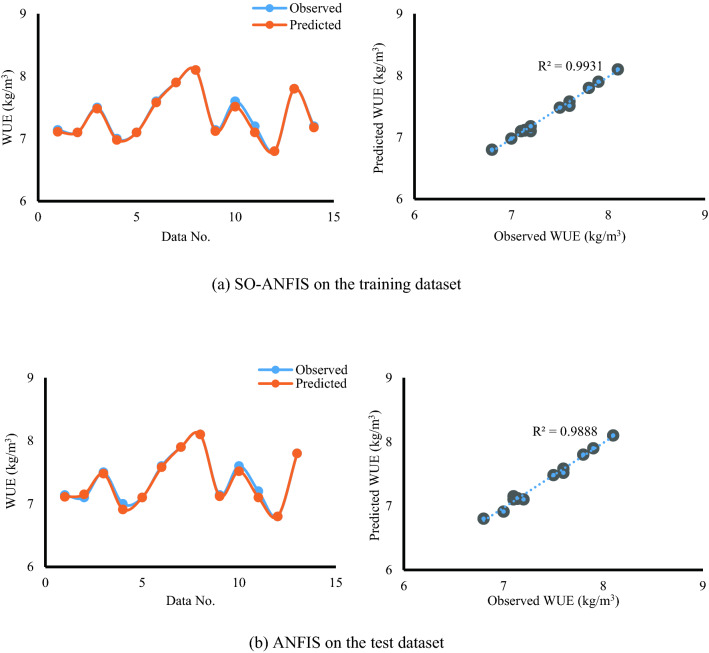
(**a**) SO-ANFIS on the training dataset, (**b**) SO- ANFIS on the test dataset. Comparison of predicted WUE with observed results in the training and test stages.

According to Figs. 11 and 12, it is clear that the yield using the SO-ANFIS hybrid method is estimated with high accuracy and is in good agreement with the observed values. Also, *ω2* modeled the yield with lower error (*RMSE* = 0.006) according to the irrigation parameter.

**Figure 13:**
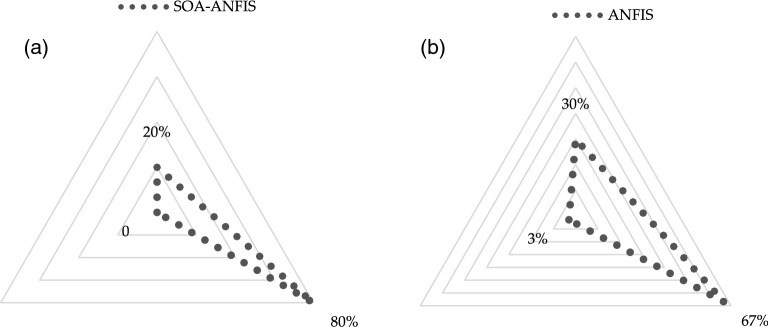
Error distribution of (**a**) SO-ANFIS and (**b**) ANFIS on the test stage.

The SO-ANFIS and ANFIS error distribution diagrams on the test stage are shown in Fig. 13. The results show that about 80% of the yield values estimated utilizing the SO-ANFIS have an error of less than 2%.

#### Comparison of SO-ANFIS with other methods

Table 9 compares the results generated by the proposed SO-ANFIS and other counterparts. The results confirm that the proposed SO-ANFIS outperformed its counterparts in estimating yield and WUE. Comparison of the results of the present study with other works shows acceptable accuracy (*R*^*2*^ = 0.988 in test stage). Compared to similar studies such as Sharifi^26^ and Prasad et al.^27^, which have evaluated the crop yield and WUE using the random forest (RF) and Gaussian process regression (GPR), the SO-ANFIS with R^2^ = 0.988 and *RMSE* = 0.006 has a better performance than the mentioned methods and can be used as a powerful method in estimating the yield and WUE.

**Table 9:** Comparison of the efficiency of SO-ANFIS model with similar methods.

Model	R^2^	RMSE	SI	δ	NSE
GPR	0.840	0.055	–	–	0.835
RF	0.690	0.045	–	–	0.687
ANFIS	0.965	0.009	0.802	0.898	0.958
SO-ANFIS	0.988	0.006	0.007	0.860	0.982

## Conclusion

In this study, the effect of *NSI* method on yield and WUE in apple orchards was investigated. The SO-ANFIS method was proposed to estimate WUE and yield in the NSI model. In the SO-ANFIS, six models were created to determine the most effective parameters in estimating WUE and yield of NSI method. The SO-ANFIS with model *ω6* generated the superior results with *R*^*2*^ = 0.988, *RMSE* = 0.006, *SI* = 0.007, *δ* = 0.860, and *NSE* = 0.982, respectively. One of the future works is to apply the SO-ANFIS method to other engineering problems to identify its strengths and weaknesses.

## Guidelines statement

All measurements and laboratory tests performed in this study are following scientific and international standards, such as soil texture determination^41^, volumetric soil moisture monitoring^42^, and water quality analysis (EPA).

## Data Availability

The data that support the findings of this study are openly available.
